# Assessing Readability of FDA-Required Labeling for Breast Implants

**DOI:** 10.1093/asjof/ojad027.009

**Published:** 2023-04-14

**Authors:** Lauren Powell, Taylor Krivanek, Sagar Deshpande, George Landis

**Affiliations:** University of Minnesota, Minneapolis, MN; University of Minnesota, Minneapolis, MN; University of Minnesota, Minneapolis, MN; University of Minnesota, Minneapolis, MN

## Abstract

**Goals/Purpose:**

In 2021, the United States (US) Food and Drug Administration (FDA) introduced regulations to improve the delivery of breast implant patient education, including new labeling requirements. The new regulations called for a patient decision checklist to be reviewed with a physician ahead of surgery. To ensure educational materials are accessible to most patients, the National Institutes of Health recommend they be written at a sixth to seventh grade level. This study aims to assess the readability of patient-facing FDA labeling for breast implants.

**Methods/Technique:**

Eleven patient information brochures and seven patient decision checklists were obtained from four breast implant company websites. An example checklist was also obtained from the FDA website. Plain text was extracted from each of the documents. Figures, tables, references, and indices were omitted. The text files were analyzed using four measures of readability validated in clinical materials: Flesch-Kincaid Grade Level, Coleman-Liau Index, Simplified Measure of Gobbledygook (SMOG), and Automated Readability Index (ARI).

**Results/Complications:**

On average, patient information brochures were 73 pages long (range: 48-92) and included 8 figures or graphics, while patient decision checklists were 5.7 pages long (range: 4-7) and included 0 figures or graphics. Mean readability scores of brochures were a Flesch-Kincaid of 12.7, Coleman-Liau Index of 13.0, SMOG of 14.0, and ARI of 12.7, with an overall mean grade level of 13.1 (13th grade). For decision checklists, results showed a Flesch-Kincaid of 14.2, Coleman-Liau Index of 13.3, SMOG of 15.6, and ARI of 14.8, with an overall grade level of 14.5 (14-15th grade). Comparatively, the sample decision checklist published by the FDA had a Flesch-Kincaid of 13.9, Coleman-Liau Index of 13.0, SMOG of 15.5, and ARI of 14.4, with an overall grade level of 14.2 (14th grade).

**Conclusion:**

Although patient decision checklists were introduced to improve accessibility of breast implant educational materials, the level at which they are written exceeds the recommendation for the average US adult by several grade levels, suggesting that patients may not fully understand current materials. While shorter than the comprehensive information brochures available, decision checklists were more difficult to read across all readability scales assessed in this study, and neither brochures nor checklists met recommended reading levels on any of the assessed scales. In order to encourage patient understanding of surgical and implant risks and promote the positive health outcomes, the FDA and breast implant companies may consider revising labeling materials.

